# The Effect of Perceptual-Cognitive Skills in College Elite Athletes: An Analysis of Differences Across Competitive Levels

**DOI:** 10.3390/sports13050141

**Published:** 2025-04-30

**Authors:** Kuo-Cheng Wu, Hui-Chun Lin, Zi-Yi Cheng, Chih-Han Chang, Jo-Ning Chang, Hsia-Ling Tai, Su-I Liu

**Affiliations:** 1 Graduate Institute of Sports Training, University of Taipei, Taipei 11153, Taiwan; erspe@go.utaipei.edu.tw (K.-C.W.); joab211222@gmail.com (H.-C.L.); britneycheng.cheng@gmail.com (Z.-Y.C.); 2 Department of Recreation and Sports Management, University of Taipei, Taipei 11153, Taiwan; goodmanroger520@yahoo.com.tw; 3 Department of Exercise and Health Sciences, University of Taipei, Taipei 11153, Taiwan; cjn320@utaipei.edu.tw; 4 Department of Physical Education, University of Taipei, Taipei 11153, Taiwan; danatai1008@go.utaipei.edu.tw

**Keywords:** sports, elite athlete, cognitive processing, reaction time, working memory

## Abstract

Background: Athletes with expertise in sports show extensive procedural and factual information, enhancing their ability to focus attention, use cues, and anticipate events. This study examined the differentiation of perceptual-cognitive skills by focusing on attentional cues, processing speed, and working memory. Methods: The component skill approach was used to assess differences in sports expertise levels using non-sport-specific cognitive measures of perceptual-cognitive skills. The study involved a total of 127 college athletes with a mean age of 20.23 years (SD = 3.08) and an average of 10.99 years of training. Among these participants, there were 43 female athletes with a mean age of 20.23 years (SD = 3.32) and 84 male athletes with a mean age of 20.22 years (SD = 2.98). We analyzed the cohort of students who did not engage in regular sports training, identifying them as the control group for our study. A multivariate analysis of variance (MANOVA) was employed to analyze the measures of the SPT and CBT perceptual-cognitive tasks, treating them as separate dependent variables. The categorization of elite levels and participants is outlined below: there are 41 semi-elite athletes, 70 competitive elite athletes, 12 successful elite athletes, and 4 world-class elite athletes. Results: There were no differences in semi-elite and competitive elite athletes’ perceptual-cognitive skills regarding visual-spatial reaction time (Wilks’ λ = 0.956, *p* > 0.05), but there was a significant difference in the working memory span (Wilks’ λ = 0.804, *p* < 0.05). Conclusions: The study reports that elite college athletes have higher working memory, which is crucial for sport performance, compared to semi-elite athletes. However, no between-group differences were observed in reaction time.

## 1. Introduction

Sports expertise refers to the ability to consistently exhibit exceptional sporting performance and consistently superior athletic performance over time [1,2,3,4]. Researchers from a variety of disciplines, including neuroscience, cognitive psychology, sport psychology, and motor skill learning, have established an interdisciplinary field dedicated to the study of ‘expertise’. Exceptional expertise can be comprehended by analyzing the cognitive and neural processes that are developed through deliberate practice [4].

To attain expert status, athletes are required to demonstrate excellence in four domains: physiological, technical, cognitive, and emotional [3]. Although it is evident that performance excellence exists, further investigation is necessary to understand the perceptual-cognitive mechanisms that contribute to expertise. Perceptual-cognitive skills involve the capacity to identify and process information, integrate it into existing knowledge, and select and execute appropriate responses [5]. For athletes to perform at their best, it is crucial to accurately perceive orientation and timing. However, both important and unimportant task details can often be overlooked owing to the abundance of visual stimuli. Athletes need to identify the most informative areas, direct their attention effectively, and quickly gather information [6].

In recent years, researchers have attempted to identify the psychological factors of excellence in sports [3]. Research suggests that elite athletes possess procedural and declarative knowledge that enables them to identify crucial information and predict events [7,8,9,10]. Furthermore, they exhibit proficiency in decision-making and the prediction of potential outcomes [2,11]. In addition, sports expertise displays higher perceptual-cognitive skills, including efficient attention allocation and cue utilization, as demonstrated in sports, highlighting the importance of acquiring perceptual skills to develop athletic expertise [12,13]. Consequently, researchers have concentrated on elucidating the processes through which athletes acquire perceptual cues [11] and understanding their superior capacity to process task-specific information [14].

Sports events require athletes to concentrate on the most relevant cues in order to achieve optimal performance and strategy. Consequently, differences between sports experts and non-sports experts were evident in sport-specific attention allocation and information acquisition measures. Further research on various sports, performance levels, and theoretical models is required to evaluate the benefits of performance and fluency for better perceptual-cognitive skill training recommendations [15].

Research indicates that exercise positively impacts cognitive function [16,17], enhancing cognitive performance through better control of motor skills and basic cognition integration. Besides exercise, specific cognitive training, especially attention [18] and working memory [19], also boosts cognitive skills. Notably, elite athletes, particularly those in interactive and technical sports, benefit similarly from both exercise and cognitive training. High performance levels in perceptual-cognitive skills through video-based task simulations are feasible [20].

Perceptual-cognitive skills in sports are typically examined through a high-performance orientation using sports-related stimuli. Studies often involve novice, sub-sports expertise athletes, and sports expertise athletes responding to visual stimuli from the sports context to recognize movement patterns or predict strike directions. Mann et al.’s [21] meta-analysis revealed that elite athletes excelled in sport-related cognitive tasks that did not include non-sport-specific stimuli, indicating the limited transferability of these skills to other domains. However, evidence suggests that team sports athletes enhance their non-sport-specific cognitive skills. Voss et al.’s [22] meta-analysis research found small to moderate effect sizes, showing that elite athletes performed better on cognitive measures than non-elite athletes, with the largest effect on message processing speed. They concluded that examining cognitive components to link sport-specific and general cognitive skills and differentiating cognitive skills into attentional cuing, processing speed, and varied attention yielded mixed results, but confirmed skill transfer from sports to general environments. Other studies have also demonstrated a positive correlation between cognitive and motor skills. In a cross-sectional study, high-skilled soccer players exhibited better executive functioning than lower-skilled players, who outperformed the normative group [23]. The study concluded that perceptual-cognitive skills may enhance performance in interceptive sports as well [24].

Athletes’ cognitive skills can be examined through two experimental psychological research methods: (i) the sports expertise performance approach and (ii) the component skill approach. The sports expertise performance approach evaluates athletes’ specific sports performance [3,21] and allows skill transfer from the field to the laboratory (proximity transfer). Research employing this methodology indicates that individuals with expertise, as opposed to those with non-sport expertise, exhibit a more comprehensive understanding of task-related information. Research using this methodology suggests that people with expertise, as opposed to those without, demonstrate a more comprehensive understanding of task-related information. They make better use of available data, encode and retrieve relevant information more efficiently, and detect and localize objects and patterns more quickly and accurately. They also make faster and more appropriate decisions using valid information [25]. The component skill approach assesses the link between basic cognitive skills and motor expertise [3,26] and determines whether athletes differ from non-athletes in perceptual-cognitive processes.

The reciprocal relationship between working memory and attention in the domain of sports has been well documented [27,28]. Athletes appear to effectively utilize attentional resources to influence movement preparation and sustain optimal performance [29]. Furley and Memmert [30] demonstrated that the current content of working memory directs athletes’ attention, positing this as the core mechanism by which attention is allocated in the deliberate pursuit of goals. Consequently, athletes can self-regulate their attentional system, enabling the conscious execution of goal-oriented movements. Working memory capacity reflects an individual’s ability to control attention [31,32,33].

Based on the above literature on cognitive ability, the present study used a component skill approach to assess differences in sports expertise using non-sport-specific cognitive measures. Further, we investigated not only interceptive but also open-skill sports. This study aimed to investigate the levels of perceptual-cognitive skills among elite athletes. We hypothesized that athletes with higher perceptual and cognitive skills would exhibit superior performance in their respective sports.

## 2. Materials and Methods

This study focused on perceptual-cognitive skills as the main research framework, and we screened out the cognitive ability-related factors that may affect athletic performance, including different sports, athletes’ levels, visual-spatial reaction time, and working memory prediction variables, to further understand the cognitive ability factors affecting the performance of motor skills.

### 2.1. Participants

A purposive sampling method [34] was used to select student athletes who participated in interceptive, strategic, and static sports as well as a control group of students who did not participate in regular sports training. Participants should be free from obvious diseases and disabilities and should sign a “consent form” before participating in the experiment. This study was approved by the University of Taipei Institutional Review Board (protocol code: UT-IRB No IRB-2020-073, 9 February 2021–2024).

Athletes and non-athletes were selected, and the athletes were asked to complete a questionnaire developed by Swann et al. [35]. The selected athletes should have had more than two years of experience in sports, and athletes with less than two years of experience were excluded.

### 2.2. Measures

Responses were given on a display measuring 1428.4 × 803.5 mm with an 8 ms response time to record reaction times. The procedures for testing spatial priming and working memory capacity are described in the Cognitive Component Test section. The perceptual-cognitive skills were assessed using the spatial priming task (SPT) [36] and the Corsi block-tapping (CBT). A total of 54 trials were run, with significant differences between the five conditions, according to Nelson [36] PEBL technical report 2013-1. The CBT serves as a measure of spatial ability. Counting span tasks assess working memory [37]. It has been used in sports [27,38]. The participants were instructed to perform the trials after comprehending the procedures individually. The test–retest reliability coefficients were 0.90 and 0.92 for the SPT and CBT, respectively.

### 2.3. Procedure

The athletic performance of individuals was assessed using a questionnaire that incorporated competitive scores for athletes, as described by Swann et al. [35]. These scores were calculated using criteria from the existing literature and were based on questions posed by Swann et al. [35] divided into two segments: competition within the athletes’ home country, and global competition. The elite status of an athlete within their home country is influenced by the country’s size and the sport’s popularity. Globally, status is determined by the number of competitors and the effort required to excel in sports. Scores were categorized as follows: 1–4 for semi-elite athletes, 4–8 for competitive elite athletes, 8–12 for successful elite athletes, and 12–16 for world-class elite athletes.

### 2.4. Data Analysis

The normality of the distribution of continuous variables was evaluated using the Kolmogorov–Smirnov (K–S) normality test. The results showed that SPT and CBT outcomes significantly deviated from the normal distribution (*p* < 0.05). Consequently, the normality assumption for the SPT and CBT distributions was rejected. As SPTs are continuous variables and normality was rejected, bootstrap MANOVA was used to examine the reaction time of the elite group. A generalized rank-order approach for nonparametric MANOVA tests on CBT data was utilized, as outlined by Thomas et al. [39].

We used MANOVA separately for the measures of the SPT and CBT tasks as dependent variables. Wilks’ lambda values for group differences in a number of dependent variables were determined [40]. Where no significant dependent variable interactions were observed, an independent *t*-test was applied to examine performance differences in perceptual-cognitive skills between the athlete groups.

Effect sizes were expressed as partial eta-squared values in MANOVA (small ≥ 0.01, medium ≥ 0.06, large ≥ 0.14), with Cohen’s d (small ≥ 0.2, medium ≥ 0.5, large ≥ 0.8) indicating mean group differences. Significance was set at 0.05 (Cohen [41]). The formula for the effect size test was first calculated using the *t*-test value for a between-subjects *t*-test and the degrees of freedom, Cohen’s d = 2t/√(df), and rYl = √(t2/(t2 + df)). All statistical levels of α were set at 0.05. An effect size (ES) test was also performed to determine the size of the differences between the groups.

## 3. Results

### 3.1. Participant Descriptive Statistics

Participants in this study were selected by intentional sampling. There were 127 college athletes (M = 20.23 years, SD = 3.08) with an average training duration of 10.99 years, 43 female athletes (M = 20.23 years, SD = 1.92), and 84 male athletes (M = 20.22 years, SD = 2.98). The athletes included 5 badminton players with 10.60 years of training duration (SD = 0.89), 28 soccer players with 10.90 years of training duration (SD = 3.14), 16 soft-tennis players with 11.50 years of training duration (SD = 3.41), 47 taekwondo athletes with 10.74 years of training duration (SD = 3.74), 16 tennis players with 10.74 years of training duration (SD = 2.88), and 15 volleyball players with 11.93 years of training (SD = 2.97). There were 44 non-athletic students, aged 18.77 years (SD = 1.32), who had not experienced sports training. The descriptive statistics of athletic sports for competitive athletes are shown in Table 1. Table 2 lists the participants’ elite levels and scores, with higher scores indicating higher elite levels.

After the classification formula, the resulting levels were as follows: 41 semi-elite athletes (32.2%, average score 2.80), 70 competitive elite athletes (55.3%, average score 5.67), 12 successful elite athletes (9.4%, average score 9.1), and 4 world-class elite athletes (3.1%, average score 14.5), representing the distribution of competitiveness levels among the athletes. As shown in Table 2, the semi-elite and elite groups comprised 41 semi-elite athletes with a percentage of 32.3% and an average competitiveness score of 2.80, and 86 elite athletes with a percentage of 67.7% and an average competitiveness score of 9.77, respectively. Elite and semi-elite athletes are listed in Table 3.

In the subsequent analysis, sports were categorized into three distinct types: intercept, strategy, and paced. The perceptual-cognitive skills associated with athletes in these categories are presented in Table 4 and Table 5. This categorization follows the framework established by the meta-analysis of Voss et al. [22]. Intercept sports include athletes participating in badminton, soft tennis, tennis, and volleyball. Strategy sports are represented by soccer athletes, whereas paced sports are exemplified by taekwondo athletes who are involved in formation competitions.

### 3.2. Perceptual-Cognitive Skills Performance of Elite Athlete Levels

In this study, perceptual-cognitive skills were assessed using a component skill approach, specifically by examining variations in spatial reaction activation and visual-spatial working memory. The results were statistically tested among elite athletes.

#### 3.2.1. Elite Levels’ Visual-Spatial Reaction Time Capability Performance

**Figure 1:**
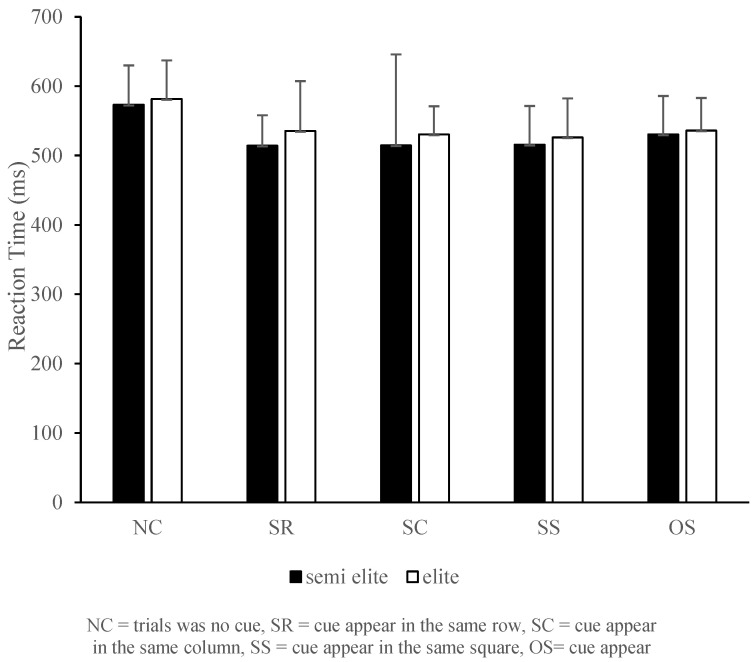
Visual-spatial reaction time in competitive semi-elite and elite college athletes.

In the statistical examination of the athletes’ perceptual-cognitive at the elite level, there were 41 athletes in the semi-elite group and 86 athletes in the elite group. In the NC performance for visual-spatial reaction time, the semi-elite group recorded 573.16 ms, SD = 79.44 ms, whereas the elite group recorded 581.56 ms, SD = 92.20. The difference between the two groups had no significant main effect in the MANOVA test, with Wilks’ λ = 0.956, F (5, 125) = 1.176, *p* > 0.05, η^2^ = 0.04, and observed power = 0.408. We further compared the differences between the two groups using independent-sample *t*-test, which showed t (126) = −0.498, *p* > 0.05, ES = 0.097. In SR performance, the semi-elite group obtained 514.06 ms, SD = 59.63 ms, and the elite group obtained 525.34 ms, SD = 65.35, with t (126) = −1.761, *p* > 0.05, ES = 0.3440. In SC performance, the semi-elite group recorded 514.54 ms, SD = 62.77 ms, and the elite group 530.28 ms, SD = 61.86, with t (126) = −1.330, *p* > 0.05, ES = 0.252. In SS performance, the semi-elite group achieved 515.59 ms, SD = 85.22 ms, and the elite group 526.01 ms, SD = 65.60, with t (126) = −0.756, *p* > 0.05, ES = 0.137. In OS performance, the semi-elite group recorded 530.26 ms, SD = 59.48 ms, and the male group recorded 520.17 ms, SD = 66.05, with t (126) = −0.741, *p* > 0.05, ES = 0.088. The above orientations show that there was no significant difference in the visual-spatial reaction time capability of the elite levels. A summary of the *t*-test of the visual-spatial reaction time of the elite levels is presented in Table 6 and Figure 1.

#### 3.2.2. Elite Athletes Working-Memory Performance

**Figure 2:**
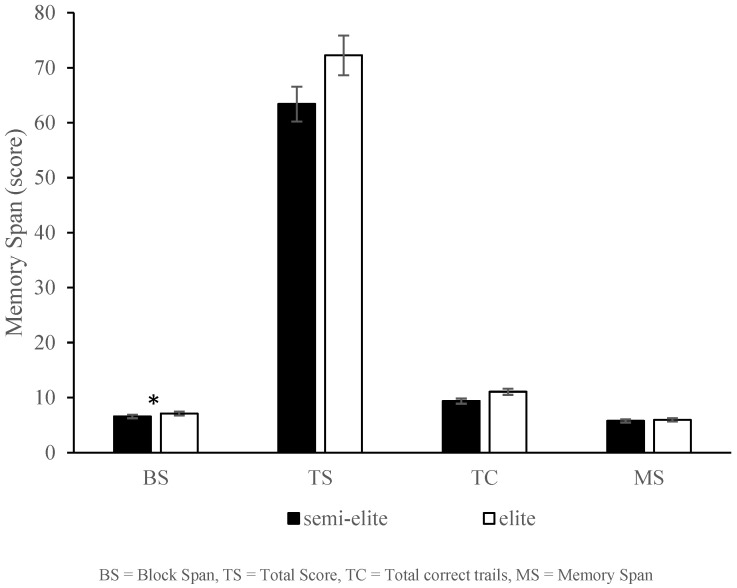
Corsi block task performance. in competitive semi-elite and elite college athletes memory span. * *p* < 0.05.

In CBT performance, assessing visuospatial short-term memory, there was a significant main effect in the MANOVA test, Wilks’ λ = 0.804, *F* (4, 125) = 8.303, *p* < 0.05, *η*^2^ = 0.19, observed power = 0.998. Athletes in the semi-elite group obtained a value of 6.56, *SD* = 1.28, and the elite group obtained a value of 7.08, SD = 1.34. Comparison of the differences between the two groups using an independent-sample *t*-test showed t (126) = 0.041, *p* < 0.05, *ES* = 0.396. In TS performance, athletes in the semi-elite group obtained a value of 63.41, *SD* = 24.23, and those in the elite group 72.27, *SD* = 24.09, with t (126) = 0.056, *p* < 0.05, *ES* = 0.366. In TC performance, the value obtained was 9.37, SD = 1.72, for the semi-elite group and 11.06, SD = 1.06, for the elite group, with t (126) = 0.333, *p* > 0.05, *ES* = 0.398. In MS performance, the value obtained by the semi-elite group was 5.75, SD = 0.793, and the value obtained by the elite group was 5.95, SD = 0.92, with t (126) = 0.925, *p* > 0.05, *ES* = 0.232. The above orientations showed that there was a significant difference in visuospatial short-term memory for the elite level, and a summary table of the *t*-tests of visuospatial short-term memory for the elite level is shown in Table 7. This result revealed that the elite group demonstrated a significant improvement in visuospatial short-term memory compared to the semi-elite group. Elite athletes have a higher cognitive capacity compared to their non-elite counterparts. A summary of the *t*-test of the working-memory performance of the elite levels is presented in Table 7 and Figure 2.

## 4. Discussion

In our study, we evaluated a variety of sports and assessed the perceptual-cognitive skills of elite college athletes at various stages, taking their gender into account. The following analysis centered on perceptual-cognitive skills, focusing specifically on visual-spatial reaction time and short-term memory in elite and semi-elite athletes. Furthermore, this analysis also examined the role of perceptual-cognitive skills in decision making, focusing on the ability to quickly process and analyze visual information and make split-second decisions in the field. Additionally, the analysis explored the relationship between perceptual-cognitive skills and performance in various sports, examining how these abilities can contribute to an athlete’s success in their respective sports.

### 4.1. Perceptual-Cognitive Skill Effects at the Elite Level

The athletes’ elite levels were determined by Swan [35], who defined elite athletes based on specific athlete characteristics. Based on the athletes’ responses to the elite athletes’ competition experiences and individual competition results, the elite athletes were categorized into semi-elite athletes and elite athletes above the competitive athletes using the calculation formula. After the calculation, there were approximately 41 semi-elite athletes and 86 competitive elite athletes.

The findings indicated no differences in elite athletes’ perceptual-cognitive skills regarding visual-spatial reaction time, but there was a significant disparity in the short-term working memory span, partially supporting the hypothesis.

### 4.2. Athletes’ Visual-Spatial Reaction Time

Visual-spatial reaction time is a fundamental requirement for athletes, particularly elite and semi-elite athletes. The absence of significant discrepancies in the visual reaction time tests between the two groups could be attributed to the crucial visual-cognitive skills of athletes in both groups. Given the extensive experience of the recruited college athletes, their reaction times aligned with those of elite performers [38]. Moreover, visual reaction time might be an acquired skill for athletes over many years of practice in a wide range of sports, including both closed- and open-skill sports [42]. It was also observed that experienced athletes had better perceived time than novice athletes in this study and other studies [43]. Research on visual reaction time in open-skill sports versus closed-skill sports also showed no differences between the two [42]. Additionally, one study found no significant elite-level effects on visual-spatial reaction time in adolescent badminton players [38].

### 4.3. Athletes’ Working Memory Performance

In this study, we examined the working memory in regulating attention and found that elite athletes outperformed semi-elite athletes in working memory performance. According to previous research, improved working memory and specialized movement skills may play a significant role in enhancing sports performance and achieving success in athletes. Recent cognitive research has highlighted the significant role of working memory in information storage, cognitive control, and attentional mechanisms [44,45,46,47]. Defined as the capacity to temporarily retain and utilize small amounts of information during tasks [45,46,47], working memory is crucial for smooth action execution [47,48]. Enhancing working memory capacity (WMC) improves adaptability to situational changes, tactical decision-making [49], focus, and efficient attention allocation [50]. As noted by Kok [35], attention and working memory interact and complement each other [51]. Elite athletes can adapt their cognitive processing modes based on the complexity of competitive situations, alternating between different cognitive processing methods rather than relying solely on unconscious autonomy [52].

These results align with those of previous studies; in the study by Williams and Ford [6], individuals with high working memory capacity were better able to adapt their tactical decisions to the situation and focus their attention on the decision-making task while ignoring extraneous auditory interference results [10,49]. According to Engle [53], a greater WMC is often associated with an increased ability to control attention. This is because WMC refers to a person’s ability in short-term memory and attentional control, i.e., the ability to hold and process multiple messages at the same time. A larger WMC enables athletes to retain and process more information concurrently, which is crucial for managing and regulating their attention. This capacity allows for more efficient resource allocation, rapid attention switching, and interference suppression, which are vital for maintaining focus during complex cognitive tasks and for effectively performing multiple tasks.

The scope of this study was confined to the amateur sports activities of college student-athletes. Although Swan et al. raised questions regarding the level of elite athletes, it is important not to extrapolate these findings to professional athletes. Moreover, due to the cross-sectional design of the study and the characteristics of the sample region and size, it is not possible to draw causal inferences regarding the development of perceptual-cognitive skills. To address this limitation, future research should incorporate longitudinal data collection methods. This approach would allow researchers to track changes in variables over time, potentially revealing causal relationships and providing a more comprehensive understanding of the subject matter. The cognitive tasks assessed included attention and working memory; however, the flexibility of cognitive function was not examined.

## 5. Conclusions

Perceptual-cognitive skills involve the ability to detect and process information, which is key to successful sporting performance. Sports experts demonstrate higher perceptual-cognitive skills, including efficient allocation of attention and use of cues, demonstrating the importance of acquiring perceptual skills in the development of sports expertise.

The study revealed that competitive elite college athletes demonstrated higher working memory and perceptual-cognitive skills compared to semi-elite athletes, which are crucial for sports performance; in other words, elite athletes are better able to adapt their cognitive processing modes to the complexity of competitive situations. They can switch between different cognitive processing methods rather than relying solely on unconscious autonomy. However, no differences were observed between the groups concerning reaction time conditions, as visual-spatial reaction time is a fundamental requirement for athletes, developed through many years of practice in a variety of sports.

The findings presented herein underscore the importance of higher-order perceptual and cognitive skills in differentiating between performance levels among athletes. To enhance performance levels in sports, it is recommended that coaches incorporate practice that focuses upon enhancing executive function and cognitive abilities. Future research should investigate more dynamic tasks to achieve a comprehensive understanding of the impact of elite athletic participation on perceptual-cognitive skills.

## Figures and Tables

**Table 1:** Descriptive statistics of athletic sports for competitive athletes.

Category	*n*	Percentage	Cumulative Percentage	Years of Training	Standard Deviation
Badminton	5	3.9	3.5	10.60	0.89
Soccer ball	28	22.1	25.6	10.90	3.14
Soft tennis	16	12.1	38.3	11.50	3.41
Taekwando	47	37.1	75.1	10.74	3.22
Tennis	16	12.5	88.5	10.74	2.88
Volleyball	15	11.8	100.0	11.93	2.97
Total	127	100.0		10.99	3.08

**Table 2:** Descriptive statistics of competitive athletes’ scores.

Elite Level	Score	Number of Athletes	Percentage	Average Score	Standard Deviation
Semi-elite	1–4	41	32.2	2.80	0.64
Competitive elite	4–8	70	55.3	5.67	1.08
Successful elite	8–12	12	9.4	9.15	1.10
World-class elite	12–16	4	3.1	14.5	1.25
Total		127	100.0		

**Table 3:** Descriptive statistics of elite athletes and semi-elite and elite scores.

	Elite Levels	Percentage	Average Competitiveness Score	Standard Deviation
Semi-elite	41	32.3	2.80	0.64
Elite	86	67.7	9.77	1.81
Total	127	88.2		

**Table 4:** Perceptual-cognitive skills: visual-spatial reaction time F-test and descriptives for intercept, strategy, and paced sports.

SPT	Types	*n*	Mean (ms)	SD
NC	Non-athlete	44	596.91	104.83
	Intercept	52	577.05	76.73
	Strategy	28	578.94	96.65
	Paced	47	558.97	68.69
SR	Non-athlete	44	519.63	56.90
	Intercept	52	534.54	62.3
	Strategy	28	525.89	64.66
	Paced	47	524.43	68.7
SC	Non-athlete	44	527.94	96.92
	Intercept	52	535.15	64.72
	Strategy	28	523.08	62.23
	Paced	47	492.45	45.18
SS	Non-athlete	44	511.89	81.67
	Intercept	52	530.77	76.75
	Strategy	28	518.74	79.03
	Paced	47	502.74	38.66
OS	Non-athlete	44	554.45	123.33
	Intercept	52	537.72	60.04
	Strategy	28	530.76	64.24
	Paced	47	521.58	67.22
	Sum	171		

NC = trials with no cue, SR = cue appears in the same row, SC = cue appears in the same column, SS = cue appears in the same square, OS = cue appears in a different row. Intercept sports: badminton, soft tennis, tennis, volleyball. Strategy sports: soccer. Paced sports: taekwondo formation competitions.

**Table 5:** Perceptual-cognitive skills: working memory F-test and descriptives for intercept, strategy, and paced sports.

Working Memory		*n*	Mean	SD
	Non-athlete	44	6.77	1.61
BS	Intercept	52	6.98	1.291
	Strategy	28	6.87	1.391
	Paced	47	7.25	1.342
	Non-athlete	44	67.86	26.34
TS	Intercept	52	69.84	21.73
	Strategy	28	68.76	24.37
	Paced	47	76.5	31.6
	Non-athlete	44	9.54	2.34
TC	Intercept	52	9.74	1.585
	Strategy	28	9.7	1.867
	Paced	47	10.13	2.363
	Non-athlete	44	5.77	1.17
MS	Intercept	52	5.87	0.786
	Strategy	28	5.85	0.928
	Paced	47	6.24	0.971
	Sum	171		

BS = block span, TS = total score, TC = total correct trials, MS = memory span. Intercept sports: badminton, soft tennis, tennis, volleyball. Strategy sports: soccer. Paced sports: Taekwondo formation competitions.

**Table 6:** Perceptual-cognitive skills: visual-spatial reaction time *t*-test for elite and semi-elite competitive athletes.

Visual-Spatial Reaction	Elite Levels	*n*	Mean (ms)	SD	t-Value
NC	semi-elite	41	573.16	79.44	−0.498
	elite	86	581.56	92.20	
SR	semi-elite	41	514.06	59.63	−1.761
	elite	86	535.34	65.35	
SC	semi-elite	41	514.54	62.77	−1.330
	elite	86	530.28	61.86	
SS	semi-elite	41	515.59	85.22	−0.756
	elite	86	526.01	65.60	
OS	semi-elite	41	530.26	66.05	−0.471
	elite	86	536.01	63.21	

NC = trials with no cue, SR = cue appears in the same row, SC = cue appears in the same column, SS = cue appears in the same square, OS = cue appears in a different row.

**Table 7:** Perceptual-cognitive skills: working memory *t*-test for competitive athletes.

Working Memory	Elite Level	*n*	Mean	SD	t-Value
BS	semi-elite	41	6.56	1.285	0.041 *
	elite	86	7.08	1.348	
TS	semi-elite	41	63.41	24.239	0.056
	elite	86	72.27	24.099	
TC	semi-elite	41	9.37	1.729	0.333
	elite	86	11.06	11.061	
MS	semi-elite	41	5.75	0.793	0.248
	elite	86	5.95	0.925	

BS = block span, TS = total score, TC = total correct trials, MS = memory span. * *p* < 0.05.

## Data Availability

Please contact the corresponding author if any additional data are required in raw form.
